# The effectiveness of rational emotive behavior therapy (REBT) and mindfulness-based intervention (MBI) on psychological, physiological and executive functions as a proxy for sports performance

**DOI:** 10.1186/s40359-023-01486-8

**Published:** 2023-12-16

**Authors:** Renátó Tóth, Martin James Turner, Joe Mannion, László Tóth

**Affiliations:** 1 School of Doctoral Studies, Hungarian University of Sports Science, Budapest, Hungary; 2 Faculty of Health and Education, Manchester Metropolitan University, Manchester, England; 3 Graduate School of Education and Psychology, Pepperdine University, Los Angeles, CA USA; 4 Teacher Training Institute, Hungarian University of Sports Science, Budapest, Hungary; 5 Department of Psychology and Sport Psychology, Hungarian University of Sport Science, Budapest, Hungary

**Keywords:** REBT, Mindfulness, Competitive anxiety, Perfectionism, Salivary cortisol, Executive functions

## Abstract

**Background:**

In the current study, we conducted a comparative analysis involving three distinct groups: one receiving group-based rational emotive behavior therapy (REBT), another undergoing a mindfulness-based intervention (MBI), and a third group serving as the control. The aim of the study was to explore the effectiveness of the two interventions on specific psychological (competitive anxiety, perfectionism, irrational beliefs), physiological (salivary cortisol levels), and neurocognitive (executive functions: working memory, inhibition, cognitive flexibility) functions in Hungarian junior ice hockey players.

**Methods:**

The participants consisted of 10 females and 36 males (N_REBT_=12, N_MINDFULNESS_=14, N_CONTROL_=20). We used questionnaires to assess competitive anxiety, perfectionism, and irrational beliefs. The components of executive functions were measured using a computerized testing system, while cortisol levels were examined through salivary samples. Prior to and after the interventions, each participant underwent all measurements, after which we conducted repeated measures ANOVA on our data.

**Results:**

We found REBT to be an effective intervention for the regulation of competitive anxiety, perfectionism, and irrational beliefs as well as for improving some components of executive functions (inhibition and cognitive flexibility). Mindfulness was found to improve athletes’ processing speed and set-shifting abilities, which are related to cognitive flexibility and metacognitive processes.

**Conclusion:**

In conclusion, we explore implications of these findings regarding how each approach is posited to enhance sports performance, using neurocognitive functions as a proxy. These findings are useful for further research and practical implications.

## Background

Performance anxiety in athletes continues to be an area of interest for research and applied practice in sport psychology. Martens and colleagues’ [1] multidimensional anxiety theory is the most widely used and accepted model of competitive anxiety in sport. This theoretical approach separates the cognitive (e.g., negative thoughts, worries) and somatic (e.g., muscle tension, increased heart rate) components of anxiety. Anxiety levels have also been shown to relate to the activity of the hypothalamic-pituitary-adrenal axis (HPA-axis), which can be detected by changes in cortisol levels [2]. However, this proposition warrants careful consideration, as stress and anxiety are distinct entities - while stress often arises from external pressures and demands on performance, anxiety tends to stem from internal apprehensions and concerns related to anticipated outcomes - and the utilization of cortisol as an indicator of anxiety may present inherent challenges. Notably, various investigations have demonstrated a positive correlation between heightened anxiety and elevated salivary cortisol levels (e.g., [2]), with salivary cortisol serving as a reliable biomarker for assessing stress levels in athletes, potentially offering a more objective insight into their anxiety levels compared to self-report questionnaires [3]. In conjunction with the correlation observed between anxiety and specific physiological indicators, research has demonstrated associations between anxiety and various psychological constructs.

One psychological factor that has been shown to exacerbate anxiety in athletes (e.g., [4]) is perfectionism whereby individuals set rigid, excessive expectations for themselves, typically accompanied by harsh self-criticism and increasing dissatisfaction even when meeting expectations [4]. In the context of sport, perfectionism can be understood as a multidimensional phenomena with two forms: adaptive (strivings) and maladaptive (concerns) perfectionism [5]. *Perfectionist strivings* are internal standards that drive the individual towards perfection whereas *perfectionist concerns* (expectations) are associated with fear of failure and negative reactions to imperfect performance [6]. Hill and colleagues [7] highlighted that despite the potential positive effects, both forms of perfectionism can have a negative impact on athletes’ mental well-being, and potentially performance. This recognition underscores the critical need for comprehensive research and intervention strategies targeting perfectionism, as the current literature lacks an adequate depth of investigation into effective treatments for athletes for mitigating its adverse effects.

Traditional psychological skills training (PST) is generally cognitive behavioural therapy (CBT) based or derived and views competitive anxiety as a negative emotional state that can lead to a drastic decline in performance if an athlete is unable to appropriately regulate it [8]. CBT is an evidence-based approach in sports psychology that focuses on identifying and modifying negative thought patterns and behaviors. The second wave of CBT focused on the identification and modification of dysfunctional thoughts and behaviors. It primarily aimed to alleviate symptoms through cognitive restructuring and behavior change, with an emphasis on treating specific mental health disorders. Rational Emotive Behavior Therapy (REBT; [9] is the original CBT, emphasizing the identification and challenging of irrational beliefs as a means to change emotional and behavioral responses. In the last decade, the use of REBT to help athletes to counter their maladaptive beliefs, thoughts, emotions, and behaviours has grown significantly in the research and practice literature [10]. Recently, it has been proposed that REBT can be applied to help athletes address perfectionism [11], and research reporting REBT with athletes has shown reductions in anxiety (e.g., [8]) and increases in performance (e.g., [14]). Fundamental of REBT is that goal-related events play a role in the development of our emotions and behaviours, and that our beliefs determine whether our emotions and behaviours are adaptive or maladaptive relative to the attainment of our goals [12]. REBT is based on the GABCDE framework [13] which states that emotional consequences (C) are the outcomes of rational or irrational beliefs (B) concerning the experienced adversity (A) related to our goals (G). An important part of the intervention is identifying irrational beliefs, which are subsequently disputed (D) and replaced by new, effective rational beliefs (E) and then reinforced [12]. *Irrational beliefs* include thoughts that are extreme, rigid, and illogical whereas *rational beliefs* are their opposite (flexible, non-extreme, logical) and contribute to the development of healthy negative emotions and productive behaviours in the service of goal achievement [12]. Irrational beliefs have been positively associated with competitive anxiety [14, 15] and perfectionism [16] in athletes. A number of studies have supported the use of REBT in sport to effectively regulate competitive anxiety by restructuring irrational beliefs [17, 18] however, the effects of REBT on perfectionism have not yet been investigated in an athlete sample. Some studies have also reported REBT to reduce cortisol levels (e.g., [19]) whereas other studies have found no significant effect (e.g., [14]).

REBT is emblematic of what is commonly called the second wave of CBT, which tends to emphasize cognitive therapy principles and methods like cognitive mediation and thought restructuring (and was preceded by the first wave’s emphasis on behavioural principles such as classical and operant conditioning). Over the last several decades, a third thematic wave of CBT has emerged that focuses on *process* or, for example, how people relate to their thought content rather than on the content itself [20]. Whereas a second wave CBT conceptualization may attribute undesirable sports performance behaviours, for example, to inadequate control of competitive anxiety or maladaptive thinking, third wave approaches are more likely to consider the ways athletes relate to such feelings and thoughts (e.g., as reified “threats” or as uncomfortable but passing benign sensations) and whether acceptance, rather than control, and refocusing attention may facilitate performance [21]. Common third wave methods like mindful awareness, phenomenology, acceptance, and values-based (versus feelings-based) behaviour help participants increase insight regarding their relationships with thoughts, feelings, and other internal and external stimuli, with a subsequent goal to modify responses to them [22]. Formal third wave programmes like mindfulness-based stress reduction (MBSR; [23]) and acceptance and commitment therapy (ACT; [24]), originally designed for physical and mental health conditions, have been adapted and integrated into sports-based mindfulness programmes such as mindfulness-acceptance-commitment (MAC; [25]), mindfulness meditation training in sport 2.0 (MMTS 2.0; [26]), and M|BODY [27]. Application areas have included personal, interpersonal (e.g., [28]), and cultural (e.g., [17]) needs in and outside sport.

Bishop and colleagues [21] proposed an operational definition of mindfulness comprised of two components: (a) self-regulation of attention (moment-by-moment observation of one’s thoughts, emotions, body sensations, and sensory experiences) and (b) a deliberate manner of relating to what is observed (non-elaborative curiosity, openness, compassion, and acceptance). Previous studies have often used a combination of different elements of mindfulness-based programs to create an adapted mindfulness intervention [29]. Results have shown such programs can reduce distress, anxiety, and improve mindfulness skills in athletes (e.g., [28]). In their review, Buhlmayer and colleagues [30] found mindfulness-based interventions can also have positive effects on certain physiological (salivary cortisol levels and immune responses) and psychological (flow, anxiety) factors in athletes as well as evidence of performance-enhancing effects. Several studies have supported an inverse relationship between cortisol and mindfulness interventions (e.g., [31]). There is less empirical evidence on the association between mindfulness and perfectionism, but Wimberley and colleagues [32], for example, found that mindfulness-based interventions may reduce maladaptive perfectionism. Research has also shown mindfulness to improve executive functions like working memory [33] and attention and cognitive flexibility [34], including in athletes [35], as well as measures of correlated neurobiology [36]. It is possible that markers of executive function can act as proxy assessments for performance in a sporting domain, which could be important because assessing performance in many sports is complicated. Indeed, in certain sports, such as ice hockey, assessing performance presents inherent challenges due to the complexity of measurement parameters and contextual variables. Several studies have shown executive functions are correlated with physical sport performance (e.g., [37]). Executive functions are neurocognitive abilities that facilitate goal-directed behavior, include and support mental skills in sport, and are associated, in part, with evolved regions of the prefrontal cortex [38]. Executive functions include three main components: working memory, inhibition, and cognitive flexibility [37]. Inhibition functions include interference (attentional) control (i.e., the ability to direct attention to relevant stimuli) and response or behavioural inhibition (i.e., the suppression of impulsive responses). Working memory is short-term, limited in capacity, and divisible into verbal and spatial-visual parts. Cognitive flexibility (often called mental set shifting) allows individuals to accurately and efficiently shift from irrelevant tasks to goal-relevant tasks [37]. Components of executive function have also been associated with competitive anxiety [39], consistent with evidence of a generally inverse relationship between activity in executive function-involved areas of the prefrontal cortex and the amygdala, which activates the HPA-axis [40].

### The present study

Most previous REBT and mindfulness research has been cross-sectional, a somewhat unsuitable method of exploring causal relationships (e.g., [13, 41]). In the present study, we used an experimental design (longitudinal) to investigate the effects of these two interventions on athletes’ competitive anxiety, perfectionism, components of executive functions, and salivary cortisol levels. One of the strengths of the study is it also included objective computer (executive function) and physiological (salivary cortisol) measures in addition to self-report questionnaires. Although the majority of mindfulness programmes have been implemented in a group setting, REBT has been dominated by individual interventions and case studies [13]. Furthermore, there is no research in sport psychology that has investigated REBT and mindfulness interventions concurrently in a group setting with a control group. The current research provided an opportunity to examine the effectiveness of the interventions separately (e.g. REBT_pre_ vs. REBT_post_) and comparing groups (REBT vs. mindfulness, REBT vs. control, mindfulness vs. control). It was hypothesised that athletes undergoing REBT would report reductions in competitive anxiety, perfectionism, irrational beliefs and show decreased cortisol levels, and improved executive function (H1). We also hypothesised that athletes participating in mindfulness would experience decrements in the level of competitive anxiety, associated cortisol levels, and improvements in the component of executive function. Although mindfulness emphasizes acceptance (rather than control or suppression) of thought content, we hypothesized increases in nonjudgement and self-compassion (facets of mindful awareness) might lead to reductions in perfectionism and irrational beliefs (H2). For the control group, we hypothesized that there would be no statistically significant alterations in any of the variables (H3), as the group did not get any intervention.

## Method

### Sample

After obtaining research ethical approval (TE-KEB/16/2022) all participants (*n* = 54) and their legal representatives were informed and gave written informed consent to take part. Female (*n* = 10) and male (*n* = 36) athletes of a Hungarian junior ice hockey club participated in the study. Eight athletes were excluded from the study due to incomplete tests, extreme outliers, and normality violations, resulting in a final sample of 46 athletes (M_age_ = 18.04, SD_age_ = 1.83). The REBT intervention included 12 (M_age_ = 18.17, SD_age_ = 1.40), the mindfulness intervention included 14 (M_age_ = 17.36, SD_age_ = 2.10) and the control group included 20 (M_age_ = 18.45, SD_age_ = 1.79) players. Each group included female and male players. We performed a priori power analysis by G*Power [42]. Large effect size (*f* = 0.41, *p* < 0.05, 1-β > 0.85) was used to calculate the preliminary sample size indicating that 30 participants are needed for mixed (within-betweens) analysis of variance (ANOVA), and if we considered within-subject separately then 24 and for between-subjects 54 athletes need to participate to achieve the appropriate effect size. When determining group sizes, we relied on recommendations from the literature and research involving similar interventions (e.g., [14, 40]). Based on these sources, the experimental group could consist of a maximum of 15 participants. Our sample size is based on these criteria.

### Procedure

**Fig. 1:**
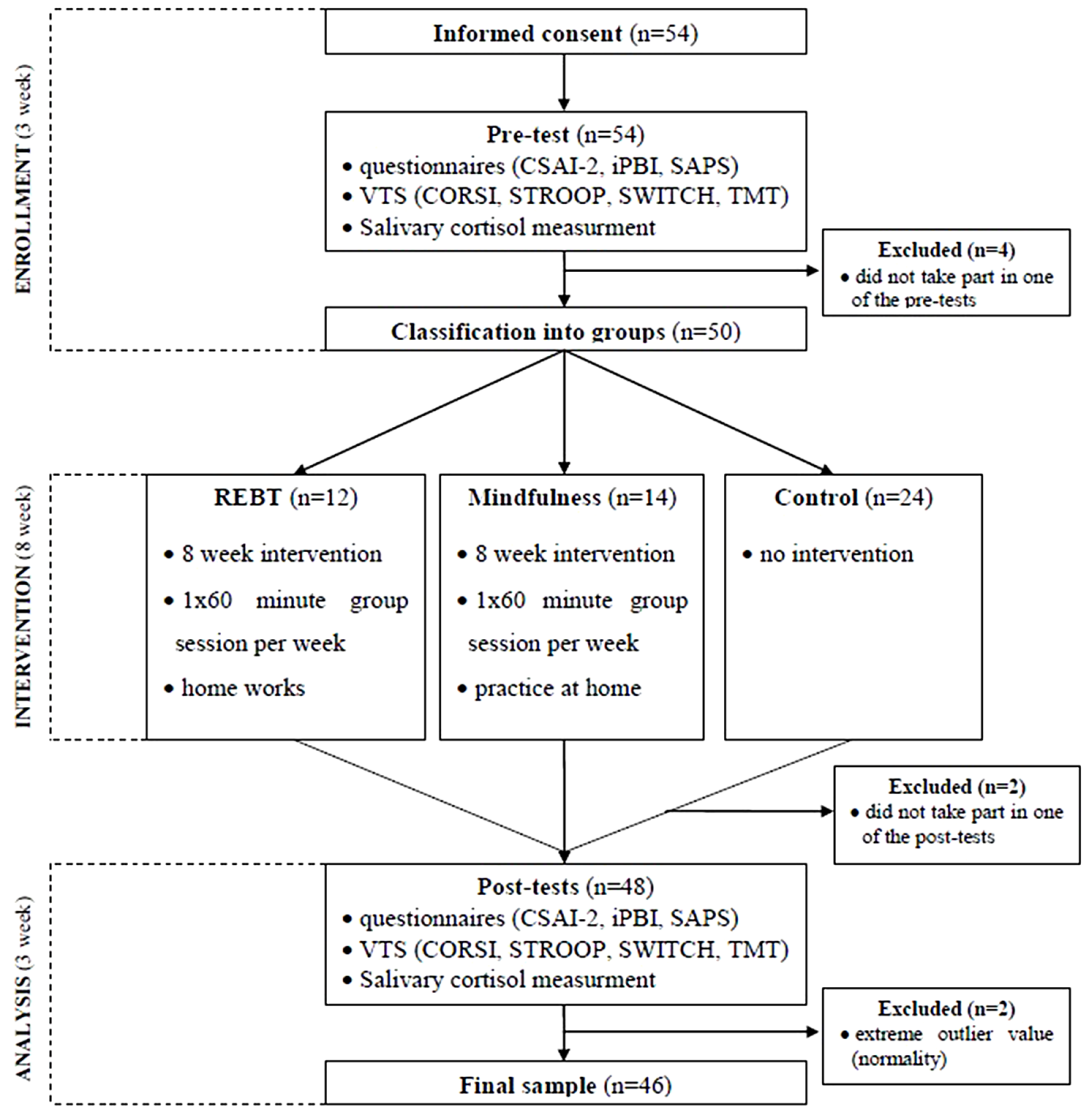
Research process

To test the effectiveness of REBT and mindfulness, we used a quasi-experimental design with pre-test and post-test time points. A limitation of previous REBT studies in sport is that most are not longitudinal and experimental (e.g., [11]; see 14 for an exception). Participants were randomly assigned to one of three groups (REBT, mindfulness, control) but there were participants who could not attend the pre-assigned group so they were placed in the control group. All measurements were conducted at a separate and appropriate location situated on the ice rink. The administration of questionnaire measurements and saliva cortisol sampling was carried out in group settings, with all participants simultaneously completing the questionnaires and providing saliva samples before and after the interventions. In the case of computerized measurements, due to the availability of only one measurement device, these were administered individually, both before and after the interventions. We attempted to limit group cross- pollination by informing participants that the topics of the group sessions were confidential and could not be shared with players from outside of their assigned group. After the research was completed, the athletes in the control group were given the opportunity to participate in the other groups and the experimental groups also offered the opportunity to participate in the other group they did not participate in. The 8-week interventions were followed by post-test measurements (see Fig. 1).

In addition to using a quasi-experimental design, the current study is also novel in that the interventions were implemented in a group format and allow for simultaneous testing of REBT and mindfulness programs. Group REBT has been used effectively in previous research (e.g., [43]), but rarely in comparison to an alternate intervention (for an exception [44]).

**Table 1:** Structure of interventions

Week	REBT		Mindfulness	
1	Education and training	Presentation of REBT approach. Teaching the GABCDE framework and inserting your own situation.	Education	Introduction to mindful awareness and the principles of mindfulness (acceptance, non-judgement and non-avoidance). Introduction to the meditation circle.
2			Self-compassion	Dealing with emotionally difficult situations, criticism and concerns about sport. **Task**: Love breathing exercises.
3	Disputation	The relationship of irrational beliefs with emotions and behaviour. Challenging and weakening irrational beliefs with evidence, logic and pragmatics.	Acceptance	Facing with the reality and the relationship between the presence and acceptance. **Task**: mindful meditation.
4			Cognitive defusion	Separation of self and thoughts. Being aware of thoughts and reactions to them and their consequences. **Task**: labelling.
5			Concentration	Consciously focusing my attention on what I need in the sport at the moment. **Task**: pyramid breathing.
6			Coping	Difficulties, failures, performance under and after pressure. Facing with negative emotions (Compassion, Humanity, Mindfulness). **Task**: Corrective imagination.
7	Replace-ment and reinforce-ment	Develop and reinforce new rational beliefs and integrate them into everyday life.	Self-control	Regulating unwanted negative thoughts and physical feelings. Connecting with your own values. **Task**: mindful stretching.
8			Summary	How has this programme helped? What exercises can you use in the context of sport.

The REBT programme was implemented by a chartered psychologist, a sport psychologist candidate who was supervised on an ongoing basis by a PhD qualified sport psychologist and Health and Care Professions Council (HCPC) registered sport and performance psychologist, an REBT trained (Advanced Practicum) professional. The REBT intervention was based on the GABCDE framework as detailed in Table 1. The REBT programme consisted of 8 group sessions and 7 homework assignments for a duration of 8 weeks and each session was one hour long. The content and scheduling of the sessions followed the main REBT intervention literature [12, 13, 41]. The mindfulness-based programme was implemented by the same person as for the REBT group. Supervision was also provided by two sports psychologists with PhD degrees, and one of them is also trained in mindfulness-based interventions. The mindfulness programme consisted of 8 group sessions and 7 home-based tasks and the duration of the sessions were also one hour (see Table 1.). We primarily based the intervention on Baltzell and Summers’ [26] Mindfulness Meditation Training in Sport 2.0 programme; however, the intervention was not identical and included other elements such as mindful stretching (e.g., [23]). We utilized a waitlist control group, who did not receive an intervention during the study. The purpose of the waitlist control group is to compare the results with the effectiveness of the experimental groups.

### Instruments

#### Executive function

Measuring the three main components of executive function, we used the Vienna Test System (VTS) which is a computer-based psychological testing system [45]. The VTS is a tool for assessing a range of ability and personality factors. It is fully standardised with automated test recording and scoring, and a practice phase following instruction to ensure that the athlete has understood the task. The VTS is also available in Hungarian language and several empirical studies have demonstrated the reliability and validity of the tests in Hungarian athletes (e.g., [46]). Participants completed the tests sequentially both before and after the interventions. The CORSI is a cube test which tests the immediate block span (IBS) and measures the capacity of spatial-visual working memory. The S1 test form used in the current study involves displaying nine irregularly distributed blocks on the screen, with a hand-shaped pointer sequentially tapping on a variable number of blocks in either the shown or reverse order. The task becomes progressively challenging as the number of blocks tapped increases by one after every three items, and the test concludes after three consecutive incorrect responses. In the results (refer to Table 2), the immediate block span (IBS) value demonstrates the average number of blocks recalled by participants in the specific group during a minimum of two distinct tasks. The test form (S1) has a good reliability index (Cronbach’s α = 0.81) [47].

The STROOP test was used to measure response inhibition [48]. The test contains a red, blue, yellow and green button, participants have to press the corresponding colour or meaning as soon as possible. The test assigns interference situations as benchmarks to these basic situations where words with different colour and meaning are evaluated on the basis of reading speed (RIT) in the first test and naming speed (NIT) in the second test. The reliability index interval (Cronbach α = 0.85–0.99) of the test form we used (S7) is high [48].

The SWITCH test was used to measure switching function, a skill that measures the flexibility of switching from one task to another [49]. During the task, the athlete is presented with bivalued stimuli which can be categorised according to two dimensions (A: shape = triangle/circle, B: clarity = grey/black). Using the keyboard (5: triangle/grey, 6: circle/black), participants select the correct response based on the task instruction. For this test, three outcomes were examined: difference in speed of response between correct responses in repeated and switching tasks (TSS), percentage of correct responses in repeated tasks (CRT), percentage of correct responses in switching tasks (CST). The test has appropriate internal consistency (McDonalds ωt = 0,76) [49].

The Trail Making Test (TMT) test has been used to measure cognitive flexibility [50]. The test consists of two parts: the first part measures processing speed (WTA) and the second part measures higher cognitive abilities such as mental flexibility (WTB). In the initial task, participants were required to employ the mouse to connect scattered numbers on the screen in ascending order from 1 to 25. Subsequently, in the second task, participants were instructed to alternate between numbers and letters, arranging them in both ascending numerical and alphabetical order (1-A-2-B…). For both tasks, the test reliability (Cronbach’s α = 0.80; 0.82) is met [50].

#### Competitive anxiety

The Competitive State Anxiety Inventory-2 (CSAI-2) questionnaire [1], adapted from the Hungarian version of the questionnaire developed by Sipos and colleagues [51], was used to measure competitive anxiety. The CSAI-2 contains 27 items, with three subscales: cognitive anxiety (CAN), somatic anxiety (SAN), and self-confidence. Each scale included 7-items which were summed and divided by seven to represent the average score for the respective groups, as depicted in Table 2. The internal consistency of the Hungarian instrument is high (Cronbach’s α = 0.75–0.85).

#### Irrational beliefs

The Irrational Performance Belief Inventory (iPBI; [52]) was used to measure irrational performance beliefs. The iPBI contains 20-items and average of them indicates the level of irrational beliefs. The translation of the iPBI into Hungarian has been used in previous studies where the instrument shows appropriate internal consistency (Cronbach’s αs = 0.70–0.89) [16].

#### Perfectionism

**.** To measure adaptive (standards) and maladaptive (discrepancy) perfectionism, we used the revised Short Almost Perfect Scale (SAPS; 53]). The Hungarian adaptation of this questionnaire was developed by Reinhardt and colleagues [54]. This scale is divided into two subscales with 4 items which measured perfectionistic standards and other 4 items which measured discrepancy. As illustrated in the second table, the 4-items for standards and the 4-items for discrepancy are separately summed and averaged to show the level of adaptive (STD) and maladaptive (DCY) perfectionism. The Hungarian version has adequate reliability and validity (Cronbach’s αs = 0.81–0.83) [54].

**Table 2:** Descriptive statistics of our results

	REBT (*n* = 12)					Mindfulness (*n* = 14)					Control (*n* = 20)				
	Pre		Post			Pre		Post			Pre		Post		
	**M**	**SD**	**M**	**SD**	**η** ^**2**^	**M**	**SD**	**M**	**SD**	**η** ^**2**^	**M**	**SD**	**M**	**SD**	**η** ^**2**^
Age	18.17	1.40	18.17	1.40	-	17.36	2.10	17.36	2.10	-	18.45	1.79	18.45	1.79	-
COR	3.02	2.38	2.45	1.18	0.05	3.31	2.97	3.16	2.03	0.01	2.75	1.55	3.53	2.34	0.12
IBS	6.75	1.14	7.00	1.21	0.04	6.79	1.05	6.79	0.98	0	6.75	1.37	6.85	1.39	0.01
RIT	**0.21**	**0.11**	**0.14**	**0.05**	**0.41**	0.18	0.07	0.21	0.19	0.02	0.21	0.11	0.20	0.11	0.07
NIT	0.13	0.06	0.12	0.08	0.04	0.10	0.06	0.11	0.05	0.08	0.11	0.05	0.11	0.04	0.02
CRT	**87.3**	**9.24**	**94.50**	**4.17**	**0.43**	95.21	3.96	95.64	4.18	0.01	92.40	10.40	93.10	9.69	0.03
CST	84.67	10.05	90.25	6.05	0.19	**91.29**	**3.63**	**94.77**	**4.00**	**0.27**	**90.7**	**8.14**	**93.00**	**7.36**	**0.23**
TSS	0.22	0.19	0.18	0.17	0.04	0.24	0.13	0.21	0.09	0.08	0.21	0.15	0.23	0.14	0.02
WTA	**15.35**	**2.24**	**13.90**	**1.63**	**0.48**	**15.96**	**1.75**	**14.53**	**2.29**	**0.28**	**16.53**	**3.03**	**16.10**	**2.59**	**0.19**
WTB	**19.56**	**4.27**	**16.43**	**3.25**	**0.52**	19.73	3.21	17.78	3.48	0.19	**21.84**	**5.10**	**19.75**	**4.37**	**0.26**
CAN	**2.41**	**0.56**	**2.13**	**0.40**	**0.41**	2.79	0.67	2.90	0.76	0.05	2.33	0.57	2.40	0.56	0.05
SAN	**1.90**	**0.40**	**1.70**	**0.46**	**0.39**	2.01	0.51	2.07	0.56	0.02	1.84	0.43	1.86	0.47	0.01
TOT	**3.63**	**0.62**	**3.30**	**0.62**	**0.37**	3.83	0.71	3.73	0.70	0.08	3.53	0.63	3.46	0.62	0.03
DCY	**6.04**	**0.65**	**5.21**	**1.07**	**0.35**	5.61	0.54	5.43	0.92	0	5.30	1.05	5.08	1.32	0.17
STD	**4.38**	**1.17**	**3.50**	**1.25**	**0.57**	4.38	1.17	4.38	1.15	0.03	4.15	1.28	4.66	1.39	0.04

Variables in bold indicate significant differences between pre and post-tests in the same group

COR: salivary cortisol level; RSS: reliable spatial span, IBS: immediate block span; RIT: reading interference tendency, NIT: naming interference tendency; CRT: correct repeated task, CST: correct switching task, TSS: task switching speed; WTA: working time part A, WTB: working time part B; CAN: cognitive anxiety, SAN: somatic anxiety; DEM: demandingness, LFT: low-frustration tolerance, AWF: awfulizing, DEP: depreciation TOT: total of irrational beliefs; DCY: discrepancy (maladaptive perfectionism), STD: standards (adaptive perfectionism)

#### Salivary cortisol

Salivary cortisol testing was performed by a qualified Hungarian company (SYNLAB Hungary Kft). During the sampling, oral swabs specially developed to measure cortisol were chewed in the athletes’ mouths for two minutes to collect enough saliva, and then the swabs were put back into the insert. Immediately after sampling, the samples were transported to the laboratory where they were stored at the appropriate temperature and analysed within 24 h. Analysis was performed using ECLIA (electrochemiluminescence immunochemistry) on a cobas immunochemical analyser [55]. Cortisol levels obtained from the athletes’ saliva were reported in nanomoles per litre (nmol/L).

## Results

Descriptive statistics and main analyses were performed using the IBM SPSS statistical analysis software. Means and standard deviations are illustrated in Table 2. The final sample data do not contain missing values and extreme outliers, the conditions of homogeneity and normality are met (*p* > 0.05). Cognitive anxiety (Cronbach’s α = 0.84), somatic anxiety (Cronbach’s α = 0.80), adaptive (Cronbach’s α = 0.78) and maladaptive (Cronbach’s α = 0.74) perfectionism scales, as well as the irrational performance beliefs inventory (Cronbach’s α = 0.89) show reliable results in the pre-tests. We found similar results in the post-tests, cognitive (Cronbach’s α = 0.86) and somatic (Cronbach’s α = 0.85) anxiety, adaptive (Cronbach’s α = 0.82) and maladaptive (Cronbach’s α = 0.81) perfectionism, and irrational beliefs (Cronbach’s α = 0.90) show good reliability values. In the main analysis repeated measures ANOVA was used to compare the pre- and post-test results (COR, CSAI-2, iPBI, SAPS, CORSI, STROOP, SWITCH, TMT) and the groups.

In the repeated measures ANOVA, the time x group main effect was tested for each target variable. The correct responses on repeated tasks (CRT) of the SWITCH test show a significant main effect between time and groups (*F* (2,43) = 5.94, *p* < 0.01, η^2^ = 0.22). The questionnaire measures also shows a significant main effect on cognitive anxiety (*F* (2,43) = 3.99, *p* = 0. 03, η^2^ = 0.16) and maladaptive perfectionism (*F* (2,43) = 3.61, *p* = 0.03, η^2^ = 0.15).

### REBT group

**Fig. 2:**
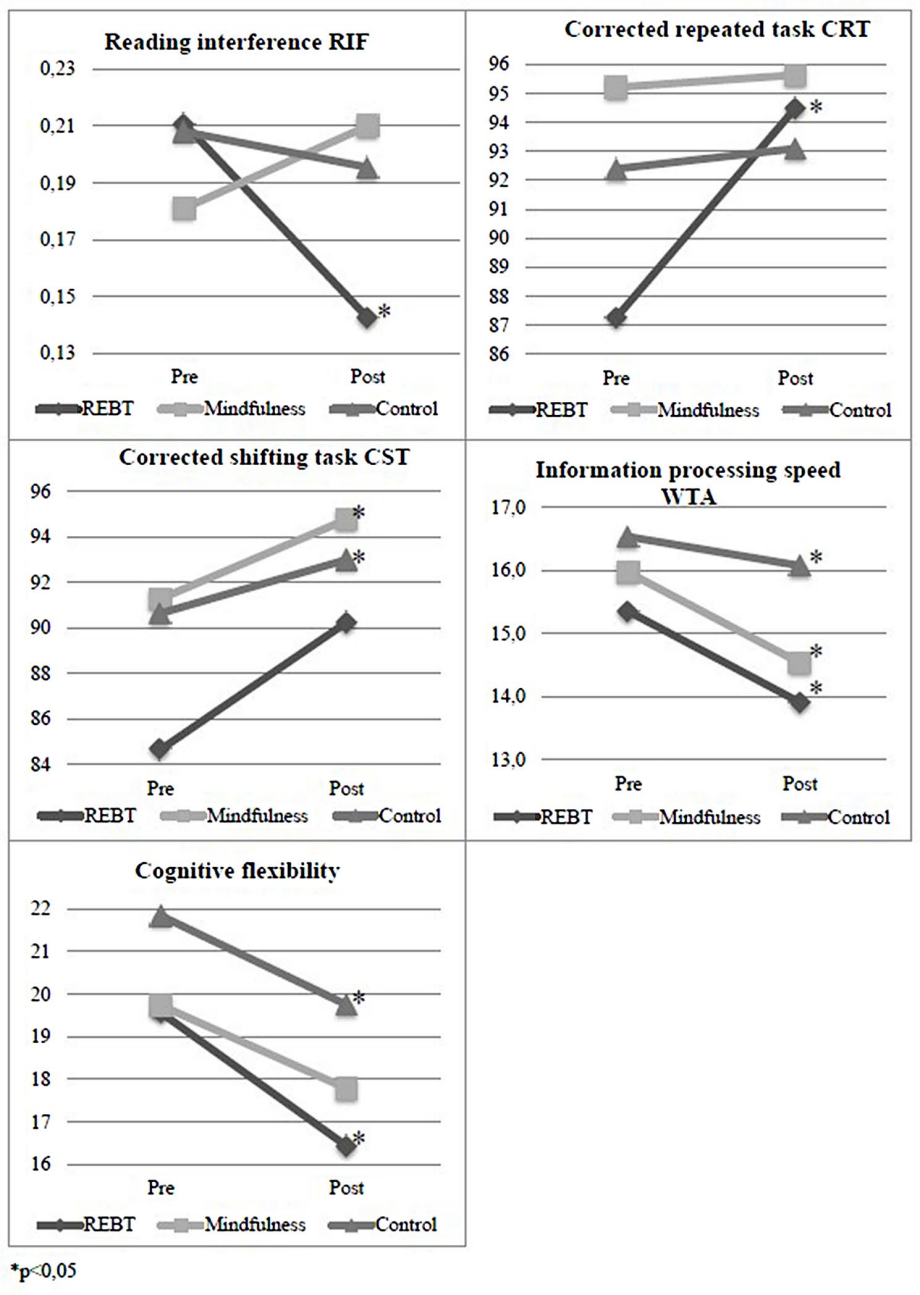
Significant differences in executive functions

Results for executive functions (see Table 2) show that athletes participating in the REBT programme show several significant differences from pre- and post-tests: reading interference tendency (*F* (1,11) = 7.53, *p* = 0.02, η^2^ = 0.41), percentage of correct repetitive tasks (*F* (1,11) = 8.34, *p* = 0.02, η^2^ = 0.43), speed of information processing (*F* (1,11) = 10.06, *p* < 0.01, η^2^ = 0.48) and cognitive flexibility (*F* (1,11) = 11.85, *p* = 0.01, η2 = 0.52). The score of reading interference tendency in the REBT group decreased from pre-test (M_pre_ = 0.21) to post-test (M_post_ = 0.14) indicating improvement. These results indicate improvement in the component of executive functions in all cases (see Fig. 2.). The cognitive flexibility task also shows a significant decrease (*F* (1,19) = 6.82, *p* = 0.02) in the control group (M_pre_ = 21.84, M_post_ = 19.75) but the effect size is higher in the REBT group (η^2^ = 0.52) than in the control group (η^2^ = 0.26). The decrease refers to time, so it took less time to complete the task (i.e., faster) which is an improvement.

**Fig. 3:**
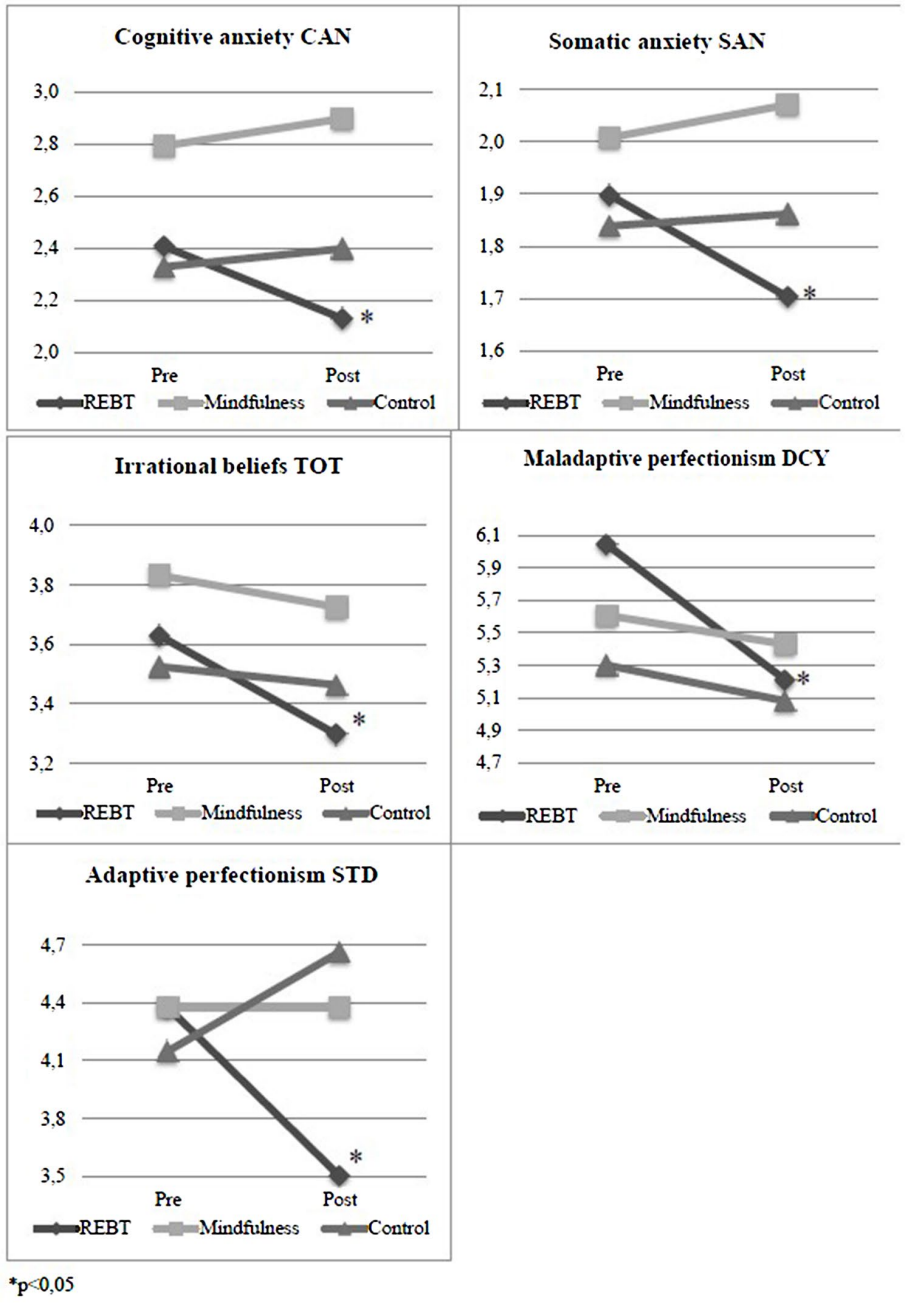
Significant differences in questionnaires

Athletes participating in the REBT program show significant differences in the questionnaire measures as well: cognitive anxiety (*F* (1,11) = 7.53, *p* = 0.02, η^2^ = 0.41) and somatic anxiety (*F* (1,11) = 6.99, *p* = 0.02, η^2^ = 0.39), irrational beliefs (*F* (1,11) = 6.43, *p* = 0.03, η^2^ = 0.37), adaptive (*F* (1,11) = 14.57, *p* < 0.01, η^2^ = 0.58) and maladaptive (*F* (1,11) = 6.00, *p* = 0.03, η2 = 0.35) perfectionism. In all cases, the significant differences indicate that the scores on these scales decreased after the interventions compared to the pre-tests (see Fig. 3).

### Mindfulness group

Based on the results of executive function (see Table 2), athletes participating in the mindfulness program show significant differences between pre- and post-tests in the percentage of correct shifting task (*F* (1,13) = 4.75, *p* = 0.05, η^2^ = 0.27) and in the speed of information processing (*F* (1,13) = 4.96, *p* = 0.04, η^2^ = 0.28). The score of correct switching tasks increased from the pre-test (M_pre_ = 91.29) to the post-test (M_post_ = 94.77) while the speed of information processing decreased (M_pre_ = 15.96, M_post_ = 14.53), indicating improvement of both variables (see Fig. 2). The correct shifting task also showed a significant increase (*F* (1,19) = 5.73, *p* = 0.03) in the control group (M_pre_ = 90.70, M_post_ = 93.00) but the effect size was higher in the mindfulness group (η2 = 0.27) than in the control group (η2 = 0.23). The information processing speed shows a significant improvement in REBT (*F* (1,11) = 10.06, *p* < 0.01), mindfulness (*F* (1,13) = 4.96, *p* = 0.04) and control groups (*F*(1,19) = 4.39, *p* = 0.05) (see Fig. 2), but the effect size is larger in the REBT (η2 = 0.48) and mindfulness (η2 = 0.28) groups than in the control group (η2 = 0.19). The results of the questionnaire measures for the mindfulness group do not show significant differences between pre- and post-tests.

## Discussion

Our results suggest levels of cognitive and somatic competitive anxiety are decreased after REBT, a common finding in REBT [10]. This finding also provides further evidence that restructuring irrational beliefs contributes to the regulation of various forms of anxiety perceived by athletes (e.g., [18]). This hypothesis is further supported by our results showing that total of irrational beliefs also decreased in participants in the REBT intervention. Furthermore, both adaptive and maladaptive perfectionism scores decreased after the intervention in the REBT group. Our results suggest that beside competitive anxiety and irrational beliefs, REBT in a group format may also help reduce perfectionism, an idea ventured by Jordana and Turner [11]. Testing executive functions, we found that all three groups (REBT, mindfulness, control) showed improvements in post-intervention measures of information processing and cognitive flexibility (TMT) compared to pre-intervention measures. The rate of improvement in information processing was highest in the REBT intervention group, and athletes in the mindfulness group also showed greater improvement than athletes in the control group. Based on these findings, it is concluded that restructuring irrational beliefs into rational beliefs and the acquisition of mindfulness skills (e.g., attentional control, acceptance, self-compassion) contribute to the athletes’ ability to absorb stimuli more quickly. Measuring cognitive flexibility, significant results were found for athletes in the REBT and control groups. The greatest improvement is shown by the ice hockey players in the REBT intervention. Taken together, these findings support our first hypothesis.

Our results also support that REBT can be used in a group setting effectively to develop certain psychological aspects of athletes such as anxiety and perfectionism. Furthermore, administering REBT in a group format can hold other key advantages for athletes. It fosters a supportive community, allowing athletes to share experiences and coping strategies. Group sessions are cost-effective and accessible, reaching a broader athlete population. Moreover, the group dynamic might simulate competition conditions somewhat, enhancing practical skill development. Also, it is more likely that the intervention is standardised across participants, because fundamentally they all received the same content, thus enhancing treatment fidelity [43].

No significant changes in salivary cortisol levels were found in any of the REBT, mindfulness or control groups of athletes, although salivary cortisol levels in the REBT and mindfulness intervention participants decreased after the interventions, while those in the control group increased. This finding reinforces previous research [19, 30, 56], with some studies demonstrating a reducing effect of the interventions on cortisol levels and others failing to reveal a significant effect. It is important to note that the structure and process of cortisol sampling is not identical in these studies, thus the development of a uniform protocol could facilitate conceptualisation of the results.

In the mindfulness and control groups, none of competitive anxiety, irrational beliefs, nor perfectionism showed significant differences between pre- and post-tests, which might relate to several factors. Mindfulness is often touted for anxiety and stress benefits, which has contributed both to its Western popularity and frequent conflation with control-based relaxation techniques, common to traditional PST [22]. Perhaps principles of mindfulness have less face validity or intuitiveness in Western cultures. Whereas REBT explicitly focuses on changing irrational beliefs, mindfulness practices focus, for example, on accepting one’s thoughts as they are (i.e., their temporary presence in awareness, not their accuracy) and *avoiding* attempts to change or suppress them, based on phenomenology (e.g., thoughts are passing cloudlike “inner sensations,” not concrete threats or equivalent to external reality). A mindful disposition often paradoxically results in decreased anxiety; however, when it does not, that is generally not considered “failure” but, instead, is also accepted with compassion and openness, frequently as a form of exposure and response prevention in more Western terms (see Birrer and colleagues [57], for an exploration of mindfulness mechanisms in sport). The desire to use mindfulness to control anxiety, though, can subvert key qualities of practice: openness and acceptance. To the contrary, increasing “windows of tolerance” for discomforts like anxiety (rather than active downregulation) is important in mindfulness (see [58]) and has been shown to facilitate performing and adapting in high pressure situations (e.g., [59]), including related to increasing resilience and the willingness to persist (e.g., [60]). Measures of changes in such tolerance levels or proxies like acceptance, though, were not included in the present study, and these differences between REBT and mindfulness might limit or confound direct comparisons. Additionally, a measure of mindfulness was not included so it is also possible relevant facets of the mindfulness group’s skills did not change or adequately improve pre- to post-intervention. Although, the mindfulness group does not show a significant improvement, the effect size is high for cognitive flexibility, confirming the results of Moynihan and colleagues [61]. This finding is also confirmed by the change found in switching functions in our sample. Athletes who participated in the mindfulness intervention showed an improvement in correct responses in a shifting task. This skill is related to cognitive flexibility, as it determines how flexible an athlete is if they are able to switch to the task that is most relevant. Of note, the mindfulness group improved their performance in these neurocognitive domains despite *not* reducing anxiety or changing thought-patterns, which is consistent with how mindfulness has been conceptualized to improve performance (i.e., by reducing disruptive effects of internal and external stimuli via acceptance rather than content-control; [57] and consistent with past mindfulness and unpleasant emotional interference task performance findings [62]. In conclusion, we could not fully support our second hypothesis but the acquisition of mindfulness skills can contribute to the acceleration of information processing and the development of cognitive flexibility.

## Conclusion

To the authors’ knowledge, the present study is the first to compare REBT and mindfulness interventions with athletes in a group setting. Regarding cultural considerations, this study also appears to be the first to investigate each intervention with Hungarian ice hockey players. Overall, the present study has revealed and confirmed that REBT can be used to cope with competitive anxiety and perfectionism and to reduce irrational beliefs. Studies on cortisol levels have shown further useful results but the impact of REBT and mindfulness interventions on physiological factors remains to be clearly explored. Examination of executive function shows that these psychological interventions may be able to improve neurocognitive performance in addition to psychological factors and potentially as a proxy for sports performance, but via different mechanisms.

The present study has some limitations. The homogeneity of the sample is an advantage of the present study, but it is also a limitation in some aspects as it restricts the conclusions that can be drawn for the whole population, although provides an opportunity to explore cultural differences. It is important to acknowledge that the use of the Competitive State Anxiety Inventory-2 (CSAI-2) as a measure in this study may raise questions regarding its appropriateness, particularly given its characterization as a state anxiety measure. While our study administered the CSAI-2 outside of a competitive environment, we recognize that the interpretation of results might appear as if it were assessing trait anxiety. We were interested in examining participants’ anxiety levels in a performance situation; however, we did not have the opportunity to measure them directly right before a competition, which is the ideal context for this instrument. Therefore, we asked them to recall how they generally feel in competitive situations (such as [17]).

The results of the present research contribute to practical applications that can serve as useful guidelines for sport psychologists. In the case of the REBT intervention, we have been able to demonstrate that the intervention can be used in a group setting to regulate competitive anxiety, perfectionism and irrational beliefs. The results of both REBT and mindfulness show that they may be effective in improving certain cognitive functions in addition to treating a number of mental health concerns but these results provide more evidence for the relevance of future research on similar topics than for their clear improvement effects on components of executive functions. Further research is needed to investigate cultural differences to ensure the applicability of mindfulness since this will allow for the development of effective programmes for specific (sport) populations in the future (for example of cultural adaptation considerations see [63]).

## Data Availability

Datasets generated and/or analysed during the current study are not publicly available as the participants did not give consent for their raw data and transcriptions to be shared with other researchers outside of the research team, but may be available from the corresponding author upon reasonable request.
